# Genomic epidemiology of the *Mycobacterium tuberculosis* complex in Fiji reveals community transmission and human *Mycobacterium bovis* infection

**DOI:** 10.3389/fmicb.2026.1933597

**Published:** 2026-08-11

**Authors:** Sakiusa C. Baleivanualala, Timothy R. M. Sahai, Joseph M. Reddy, Takoi Ciusia, Shayal S. Sharma, Yugen Miyahara, Emosi Bayanivalu, Luse Buinimasi, Robert Day, James E. Ussher, Mike Kama, Htin Lin Aung

**Affiliations:** 1 Department of Microbiology and Immunology, Faculty of Biomedical Sciences, Division of Health Sciences, University of Otago, Dunedin, New Zealand; 2 College of Medicine, Nursing and Health Science, Fiji National University, Suva, Fiji; 3 Maurice Wilkins Centre for Molecular Biodiscovery, University of Auckland, Auckland, New Zealand; 4 Ministry of Health and Medical Services, Suva, Fiji; 5 Otago Genomics Facility, University of Otago, Dunedin, New Zealand; 6 Awanui Labs, Dunedin Hospital, Dunedin, New Zealand

**Keywords:** Fiji, *Mycobacterium bovis*, *Mycobacterium tuberculosis*, treatment outcome, tuberculosis, WGS

## Abstract

**Introduction:**

Tuberculosis (TB) remains a major public health concern in Fiji, but genomic data on the circulating *Mycobacterium tuberculosis* complex (MTBC) are limited.

**Methods:**

We analysed 65 MTBC isolates collected during 2019–2022 using whole-genome sequencing to characterize lineage distribution, drug resistance, and transmission patterns.

**Results:**

Of the isolates, 62 (95.4%) were *Mycobacterium tuberculosis* and 3 (4.6%) were Mycobacterium bovis. Lineage 4 was the most common (29/62, 46.8%). Genomic analysis identified 12 transmission clusters, with epidemiological links involving households, schools, and neighbouring communities. Most MTBC isolates (57/65, 87.7%) were susceptible to first-line anti-TB drugs; however, 8 (12.3%) carried resistance-associated mutations in *katG*, *rpoB*, *rrs*, or *pncA*. Treatment outcomes indicated that 20 (30.8%) patients were lost to follow-up.

**Discussion:**

Detection of *M. bovis* suggests possible zoonotic transmission and highlights limitations of routine diagnostics. These findings indicate diverse MTBC lineages and localized transmission networks, supporting integration of genomic surveillance into TB control efforts in Fiji.

## 1 Introduction

Tuberculosis (TB), caused by *Mycobacterium tuberculosis* (Mtb), remains among the top 10 causes of death globally and is the leading cause of death from a single infectious agent (Bhargava and Bhargava, 2020). According to the World Health Organization (WHO), 10.7 million people developed TB in 2024, and an estimated 1.23 million died from the disease; approximately 87% of all cases occurred in 30 high TB burden countries, primarily in low- and middle-income regions (Estaji et al., 2024). Despite decades of global control efforts, TB continues to impose a substantial burden on health systems, particularly in resource-limited settings. Understanding the transmission dynamics, population structure, and drug resistance patterns of circulating Mtb strains is critical for strengthening TB control strategies and achieving the goals of the WHO End TB Strategy.

Advances in genomic technologies have transformed TB surveillance and epidemiology. Whole genome sequencing (WGS) has been increasingly used to identify outbreak clusters, detect drug resistance mutations and characterize previously unrecognized transmission events (Nyunt et al., 2016; Mulholland et al., 2022; Mulholland et al., 2019). Moreover, WGS enables classification of MTBC strains into distinct phylogenetic lineages, which differ in geographic distribution, transmissibility, and association with drug resistance (Micheni et al., 2021; Diriba et al., 2021). Integration of genomic, epidemiological, and clinical data provides valuable insights into TB transmission and guides targeted public health interventions (Koleske et al., 2023; Li et al., 2024). However, implementation of genomic surveillance remains limited in many low resource settings due to financial constraints, logistical challenges, and limited bioinformatics capacity.

Fiji, a Pacific Island nation with a population of approximately 900,000, has recently transitioned from an upper moderate TB burden setting to a TB endemic setting, with WHO estimates reporting an incidence of approximately 72 cases per 100,000 population (World Health Organization, 2025). TB diagnosis and treatment services are coordinated through the national TB programme, with culture confirmation and reference testing conducted at the Daulako Mycobacterium Reference Laboratory located at Twomey Hospital in Suva (Ragonnet et al., 2019). Although routine diagnostic tools such as smear microscopy and GeneXpert are widely used, genomic characterization of circulating MTBC strains has not previously been undertaken in Fiji. The first confirmed case of multidrug resistant (MDR) Mtb was reported in 2016, but no sustained transmission of resistant strains has been documented (Yanagawa et al., 2023). While recent national reports suggest improvements in treatment outcomes and progress toward WHO End TB Strategy targets, important gaps remain in understanding the molecular epidemiology and transmission patterns of TB in the country (Ragonnet et al., 2019). In particular, the diversity of MTBC circulating in Fiji, the presence of potential transmission clusters, and the genomic basis of drug resistance remain largely unknown.

This study, therefore, applied WGS to MTBC isolates collected at the Fiji national tuberculosis reference laboratory to characterize lineage distribution, phylogenetic relationships, drug-resistance mutations, and transmission dynamics of circulating MTBC strains in Fiji.

## 2 Materials and methods

### 2.1 Setting and study design

We conducted a retrospective descriptive study at Twomey Hospital, the national referral centre for TB diagnosis and treatment in Fiji. Fiji’s health system is organized into four administrative divisions: Central, Western, Northern, and Eastern (Supplementary Figure S1; Phelan et al., 2019). Specimens from patients with suspected or bacteriologically confirmed TB from all divisions are referred to the Daulako Mycobacterium Reference Laboratory (DMRL), located within Twomey Hospital, for mycobacterial culture, reference testing, and storage of culture-positive isolates.

We included a convenience sample of non-duplicate bacteriologically confirmed MTBC isolates achieved at the Twomey Hospital during 2019, 2021, and 2022. No isolates were collected in 2020 because of disruptions associated with the COVID-19 pandemic, including diversion of laboratory resources and reduced diagnostic capacity. Isolates were selected opportunistically from archived specimen available at the DMRL; no formal random sampling strategy was applied.

### 2.2 Laboratory procedures

Stored isolates were re-cultured using the Mycobacterial Growth Indicator Tube 960 system (BD Diagnostics) at 37 °C for 4 weeks (Siddiqi et al., 2012). Positive culture was selected for WGS. DNA was extracted from the supernatant using a QIAamp DNA Mini Kit (Qiagen, Hilden, Germany) according to the manufacturer’s instructions. Purified DNA was shipped to the University of Otago, Dunedin, New Zealand, for whole-genome sequencing and downstream analysis.

### 2.3 Whole genome sequencing and analysis

DNA quality and quantity were assessed using Qubit 4.0 fluorometry (Invitrogen, Thermo Fisher Scientific, Singapore) and Nanodrop spectrophotometry (ThermoFisher Scientific, MA, USA). Sequencing libraries were prepared following the Illumina DNA Prep (M) Tagmentation protocol kit (Illumina, San Diego, CA, USA), with each sample uniquely indexed. Libraries were normalized and pooled and sequenced on an Illumina NextSeq 2000 (P1 reagents), to generate 150 base pair reads. All sequencing data from this study were deposited into the NCBI sequence read archive (accession number PRJNA1494897).

### 2.4 Genomic sequence data analysis

Raw sequencing reads were analysed using TB Profiler (v6.0.0) to assign Mtb lineages and detect drug resistance-associated mutations (Phelan et al., 2019). Resistance predictions were based on the curated tbdb mutation database implemented in TB-Profiler, which incorporates mutations from the WHO catalogue of mutations in and their association with drug Resistance (Id et al., 2022; World Health Organization, 2023). Subsequently, reads were processed using the Snippy pipeline (v4.6.0) for read alignment, variant calling, and generation of a core genome single nucleotide polymorphism (SNP) alignment (Phelan et al., 2019; GitHub, 2023). Reads were aligned to the Mtb H37Rv reference genome (GenBank accession NC_000962.3). High-quality SNPs were retained using default filtering parameters and low-confidence sites were excluded.

The resulting alignment was further processed using Gubbins (Genealogies Unbiased By recomBinations In Nucleotide Sequences) to identify and mask recombinant regions prior to phylogenetic analysis (Croucher et al., 2015; Didelot and Wilson, 2015). Phylogenetic relationships were inferred from the recombination-filtered core SNP alignment. Genetic clustering of isolates was determined using Fast hierarchical Bayesian analysis of population structure (fastBAPS; Tonkin-Hill et al., 2019). Phylogenetic trees were visualized and annotated using the Interactive Tree of Life (iTOL; v7) platform (Letunic and Bork, 2024). Lineage and sublineage assignments generated by TB-Profiler, based on lineage-defining canonical SNPs in its curated database, were supported by phylogenetic clustering with the reference genomes. Pairwise core SNP distances were interpreted using commonly applied thresholds for Mtb genomic epidemiology. Isolates differing by 0–5 SNPs were considered consistent with recent or highly likely transmission, those differing by 6–12 SNPs were interpreted as possible transmission, and isolates differing by >12 SNPs were considered unlikely to represent recent transmission (Walker et al., 2013; Yang et al., 2017; Stimson et al., 2019). Data visualization of Mtb lineage and sublineage distributions was performed in RStudio (version 4.4.2; R Foundation for Statistical Computing, Vienna, Austria) using the ggplot2 package (version 4.0.1).

### 2.5 Clinical data analysis

Data was obtained from DMRL registers and patient records at the Twomey hospital and the patient information system (PATIS), and national TB programme contact tracing reports, where available. Clinical data, including patient demographics, specimen collection dates, specimen type and collection site, residential address, laboratory diagnosis, treatment history, treatment regimen, and treatment outcomes, and documented epidemiological contacts, were integrated with genomic data for analysis. Case was defined as a laboratory-confirmed TB patient. Drug-resistance classification was based on genotypic resistance prediction derived from WGS, with rifampicin resistance additionally determined using GeneXpert MTB/RIF where available. Resistance categories were assigned according to WHO definitions (World Health Organization, 2023). Potential epidemiological links were determined by integrating the genomic data with patient metadata, including geographic division, residential location, documented contact investigations, and other available clinical and epidemiological information. Given the retrospective nature of the study, epidemiological information was incomplete for some cases, limiting confirmation of epidemiological links for all genomic clusters.

This study was approved by the College Health Research Ethics Committee, Fiji National University and the Human Ethics Committee (Health) at the University of Otago (183.20 and H20/174).

## 3 Results

### 3.1 Distribution of laboratory-confirmed TB isolates in Fiji

Of the 612 laboratory-confirmed TB cases reported during the study period, 182 (29.7%) were from 2019, 194 (31.7%) from 2021, and 236 (38.6%) from 2022. Of these, 65 (10.6%) were available for analysis. Of the 65 cases, 37 (56.9%) were from the Central Division, 15 (23.1%) from the Western Division, 5 (7.7%) from the Northern Division, and 8 (12.3%) from the Eastern Division. Of the 65 cases, the median (range) age was 35 (2–79) years; 32 (49.2%) were male; 53 (81.6%) were i-Taukei (indigenous Fijians); and six (9.2%) each were Fijians of Indian descent and of other ethnic backgrounds (Table 1).

**Table 1:** Distribution and characteristics of laboratory-confirmed MTBC isolates in Fiji by year, 2019, 2021–2022 (*n* = 65).

Variable	2019	2021	2022	Total
*Mycobacterium tuberculosis*	32 (51.6%)	11 (17.7%)	19 (30.6%)	62 (95.4%)
*Mycobacterium bovis*	2 (66.7%)	1 (33.3%)	–	3 (4.6%)
Geographic division				
Central	19 (51.4%)	7 (18.9%)	11 (29.7%)	37 (56.9%)
Western	7 (46.7%)	5 (33.3%)	3 (20.0%)	15 (23.1%)
Northern	4 (80.0%)	–	1 (20.0%)	5 (7.7%)
Eastern	4 (50.0%)	–	4 (50.0%)	8 (12.3%)
Sex				
Male	15 (46.9%)	9 (28.1%)	8 (25.0%)	32 (49.2%)
Female	19 (57.6%)	3 (9.1%)	11 (33.3%)	33 (50.8%)
Age, years				
1–14	2 (66.7%)	–	1 (33.3%)	3 (4.6%)
15–29	9 (40.9%)	9 (40.9%)	4 (18.2%)	22 (33.8%)
30–44	10 (62.5%)	–	6 (37.5%)	16 (24.6%)
45–59	6 (60.0%)	2 (20.0%)	2 (20.0%)	10 (15.4%)
60–74	6 (46.2%)	1 (7.7%)	6 (46.2%)	13 (20.0%)
75–89	1 (100%)	–	–	1 (1.5%)
Ethnicity				
i-Taukei	29 (54.7%)	9 (17.0%)	15 (28.3%)	53 (81.5%)
Fijian of Indian descent	3 (50.0%)	1 (16.7%)	2 (33.3%)	6 (9.2%)
Other	2 (33.3%)	2 (33.3%)	2 (33.3%)	6 (9.2%)
Case classification				
New case^*^	34 (53.1%)	12 (18.8%)	18 (28.1%)	64 (98.5%)
Relapsed TB	–	–	1 (100%)	1 (1.5%)
Treatment regimen				
Standard 6-month regimen^+^	34 (54.0%)	11 (17.5%)	18 (28.6%)	63 (97.0%)
Re-initiated 8-month regimen^#^	–	1 (100.0%)		1 (1.5%)
Rifampicin-resistant regimen^^^	–	–	1 (100.0%)	1 (1.5%)
Treatment outcome				
Cured	21 (55.3%)	8 (21.1%)	9 (23.7%)	38 (58.5%)
Lost to follow-up	8 (40.0%)	3 (15.0%)	9 (45.0%)	20 (30.8%)
Deceased	5 (71.4%)	1 (14.3%)	1 (14.3%)	7 (10.8%)

* New case - A newly registered episode of tuberculosis in a patient who has never received anti-tuberculosis treatment or who has previously taken anti-tuberculosis medicines for less than 1 month.

+ Standard 6-month regimen refers to 2 months of HRZE followed by 4 months of HR.

# Re-initiated regimen refers to extended or modified treatment given to previously treated cases.

^ Rifampicin-resistant regimen refers to second-line treatment for rifampicin-resistant tuberculosis. Treatment appropriateness is based on genotypic resistance testing.

HRZE: isoniazid, rifampicin, pyrazinamide, ethambutol; HR: isoniazid, rifampicin. Treatment outcomes are defined according to the national tuberculosis programme guidelines.

### 3.2 MTBC population diversity

**Figure 1:**
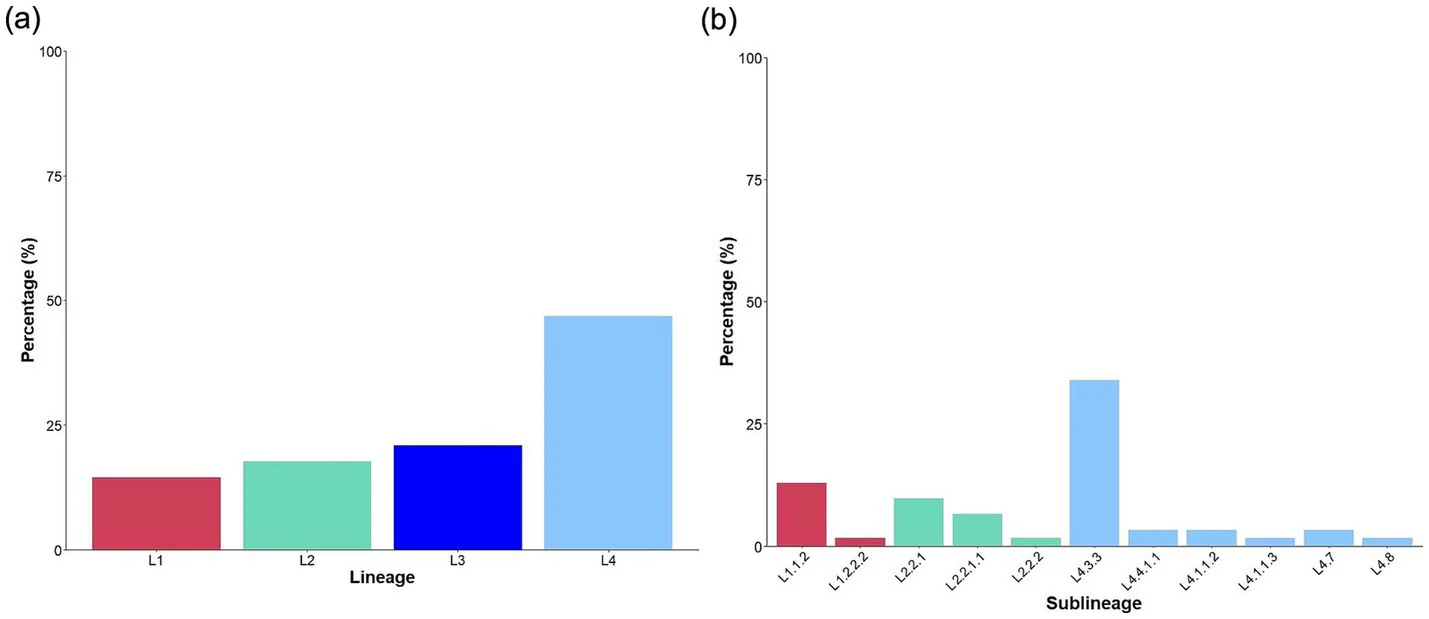
Distribution of Mtb lineage and sublineage of Mtb in Fiji, 2019, 2021–2022. **(a)** Percentage of Mtb isolates by major lineage (L1 - L4). **(b)** Distribution of Mtb sublineages within each lineage. Data are based on WGS of clinical isolates (*n* = 62) collected during the study period.

Of the 65 isolates, 62 (95.4%) were Mtb and three (4.6%) were *M. bovis*. Of the 62 Mtb isolates, 29 (46.8%) belonged to L4 (Euro-American lineage), 13 (21.0%) to L3 (East African Indian lineage), 11 (17.7%) to L2 (East Asian lineage), and nine (14.5%) to L1 (Indo-Oceanic lineage; Figure 1a).

Of the 62 Mtb isolates, sublineage L4.3.3 was the most common, accounting for 21 (33.9%) isolates, followed by L1.1.2 with 8 (12.9%), L2.2.1 with 6 (9.7%), and L2.2.1.1 with 4 (6.5%) isolates. Other sublineages included L4.4.1.1, L4.1.1.2, and L4.7 with 2 (3.2%) isolates each, while L1.2.2.2, L2.2.2, L4.1.1.3, and L4.8 were represented by 1 (1.6%) isolate each (Figure 1b).

### 3.3 Genomic and epidemiologic linkages among MTBC isolates from Fiji

**Figure 2:**
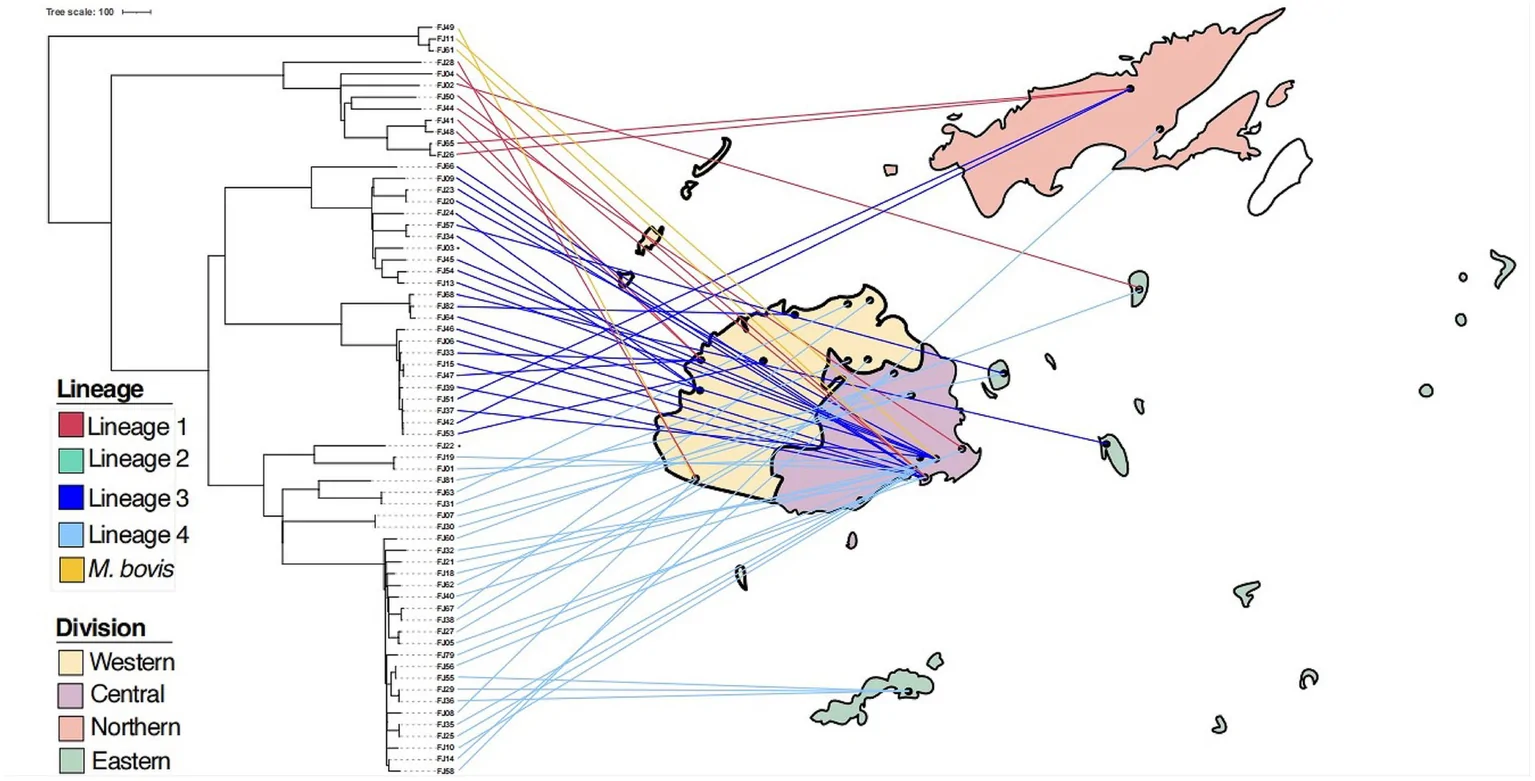
Phylogenetic tree and spatial distribution of *Mycobacterium tuberculosis* complex isolates from Fiji, 2019, 2021–22. The phylogenetic tree (left) was constructed from core genome SNP alignment of 65 isolates using the *Mtb* H37Rv reference genome (GenBank accession number NC_000962.3). Isolates are coloured according to lineage. The map (right) shows the geographic distribution of isolates across Fiji, with colours corresponding to lineages and locations based on patient residential divisions.

Phylogenetic analysis of 65 MTBC isolates demonstrated substantial genetic diversity across the four major Mtb lineages (L1 - L4) and *M. bovis* in Fiji across four administrative divisions (Figure 2). A total of 12 genomic clusters were identified among the Mtb, using SNP distances of less than 12.

**Figure 3:**
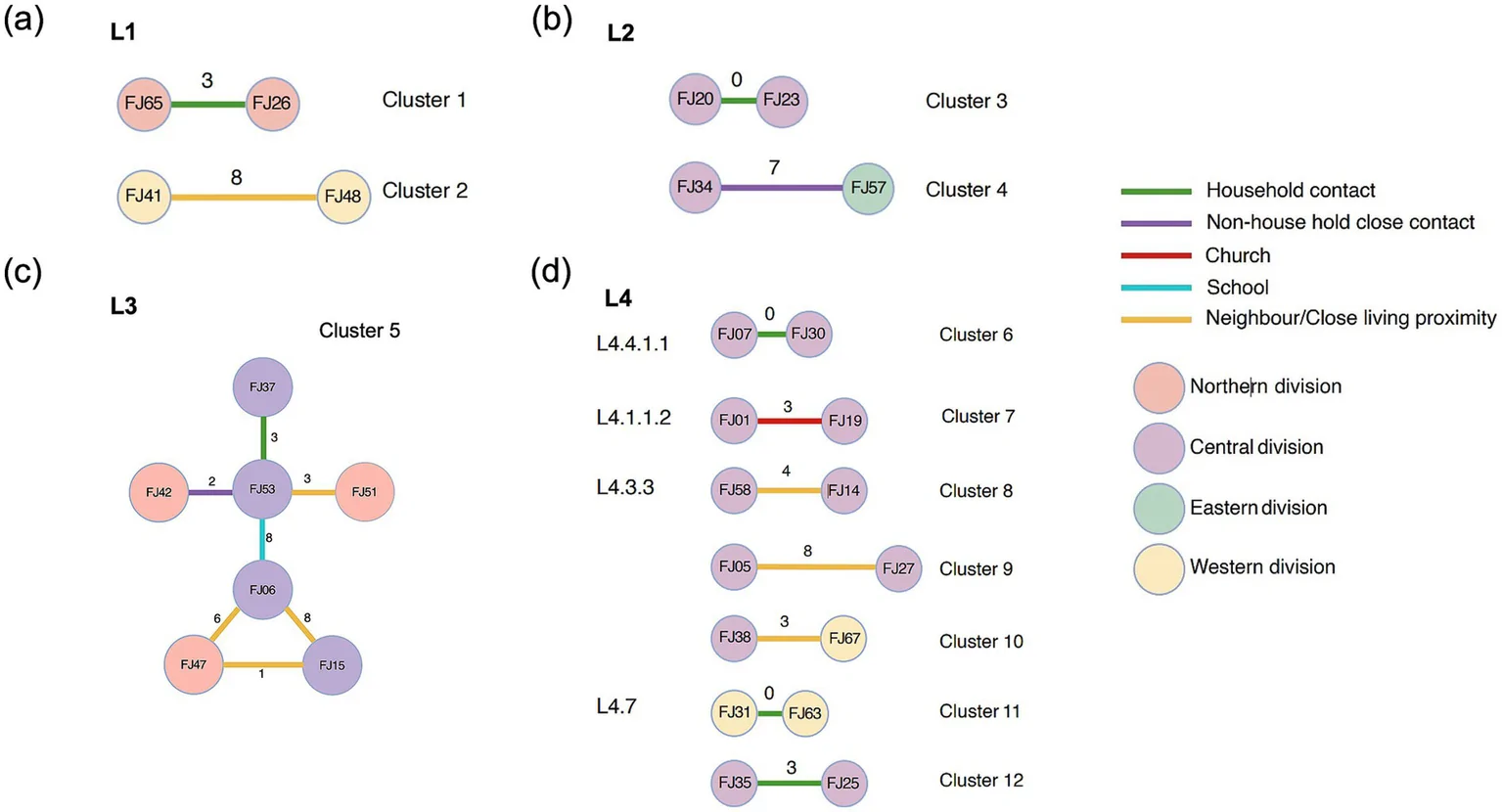
SNP-based clustering of Mtb isolates from Fiji, 2019, 2021–2022. Panels represent: **(a)** Lineage 1 (L1), **(b)** Lineage 2 (L2), **(c)** Lineage 3 (L3), and **(d)** Lineage 4 (L4). The panel presents a pairwise SNP distance network highlighting genomic clusters. Nodes represent individual isolates labelled by isolate ID, with node colours indicating the geographic division of origin. Connections between nodes represent genomic clustering based on pairwise core genome SNP) distances. Numbers shown on connecting lines indicate the number of SNP distance between linked isolates. Epidemiological links were inferred from available patient metadata, including household, community, school, church, and travel-associated contacts. The network illustrates closely related transmission clusters and highlights potential transmission pathways across social and geographic settings.

Within L1, two clusters were identified involving four (44.4%) of nine isolates (Figure 3a). FJ26 and FJ65 differed by 3 SNPs and originated from the same household in the Northern Division. FJ48 and FJ41 differed by 8 SNPs and were both from the Western Division. Within L2, two clusters (cluster 3 and 4) were identified involving 4 of 11 (36.4%) isolates (Figure 3b). FJ20 and FJ23 were identical (0 SNPs) and occurred in a household in the Central Division. FJ34 and FJ57 differed by 7 SNPs and were from patients in the Central and Eastern Divisions who were reported to be family friends and to visit each other.

Within L3, phylogenetic analysis identified a single genomic cluster comprising seven isolates (61.5%), with pairwise distances of ≤12 SNPs (Figure 3c). FJ37 and FJ53, both from the Central Division, differed by 3 SNPs and were identified as social friend contact. FJ42 from the Northern Division, who was the family relative of FJ53 differed by 2 SNPs. FJ51 from the Northern Division differed by 3 SNPs from FJ53; notably, FJ51 frequently travelled to the Central Division and resided in a neighbouring household to the FJ53 case. FJ06 and FJ53 from the Central Division differed by 8 SNPs and were linked through a school setting. FJ06 differed by 6 SNPs from FJ47 from the Northern Division, with both individuals reported to be friends and socially connected. FJ15 from the Central Division and FJ47 from the Northern Division differed by 1 SNP. Although residing in different divisions, FJ47 was a close contact of FJ06 and lived in close proximity to FJ15.

Within L4, seven genomic clusters involving 14 (48.3%) of the 28 isolates were identified (Figure 3d). Three clusters belonged to sublineage L4.3.3, two to L4.7 and one each to L4.1.1.2, L4.4.1.1. Clusters with clear epidemiological links were observed. In L4.4.1.1, FJ07 and FJ30 were genomically identical (0 SNPs) and linked through a household contact in the Central Division. In L4.1.1.2, FJ01 and FJ19 differed by 3 SNPs and were associated with a shared church setting in the Central Division. Within L4.3.3, three clusters were identified. FJ58 and FJ14 differed by 4 SNPs, and FJ05 and FJ27 by 8 SNPs; all patients resided in the Central Division. Epidemiological investigations further showed that FJ05 and FJ27 originated from the same village. FJ38 and FJ67 differed by 3 SNPs, with patients residing in neighbouring villages at the border of the Central and Western Divisions. In L4.7, FJ31 and FJ63 were also identical (0 SNPs) and linked through a household contact in the Western Division. Another cluster comprised patients (FJ35 and FJ25, separated by 3 SNPs) from the same household in the Central Division.

### 3.4 Genotypic drug resistance profile

Of the 65 isolates, 57 (87.7%) were predicted to be fully susceptible, five (7.7%) were predicted to be resistant to one drug, one (1.5%) was predicted to be resistant to two drugs, and two (3.1%) were predicted to be resistant to three drugs (Supplementary Table S1).

Of the nine L1 isolates, one (11.1%) isolate carried a *rpoB* (H445L) mutation conferring resistance to rifampicin. Of the 11 L2 isolates, one (9.1%) carried a *rpoB* (H445L) mutation conferring resistance to rifampicin, while two (18.2%) carried *katG* (S315T), *pncA* (G141P), and *rrs* (A514C) mutations conferring resistance to isoniazid, pyrazinamide, and streptomycin, respectively. All 13 L3 isolates were predicted to be fully susceptible. Of the 29 L4 isolates, one (3.4%) L4.3.3 isolate carried an *inhA* promoter mutation (C-777 T) conferring resistance to isoniazid and ethionamide. All three *M. bovis* isolates demonstrated predicted drug resistance, haboring, the *pncA* mutation conferring pyrazinamide resistance. No known mutations conferring resistance to new and repurposed drugs bedaquiline, pretomanid and linezolid were detected.

### 3.5 Treatment regimens and clinical outcomes

Of the 65 cases, 64 (98.5%) were newly registered cases, while one (1.5%) was a relapsed case. Sixty-three (96.9%) patients received the standard six-month first-line TB regimen, consisting of an intensive phase of isoniazid, rifampicin, pyrazinamide, and ethambutol for 2 months followed by isoniazid and rifampicin for 4 months (2HRZE/4HR). One (1.5%) patient received a re-initiated eight-month regimen, and one (1.5%) patient infected with rifampicin-resistant Mtb received a second-line regimen (Supplementary Table S1).

Among the 63 cases treated with the standard first-line regimen (2HRZE/4HR), two (3.2%) were infected with Mtb strains predicted to be resistant to three first-line drugs, one (1.6%) with a strain predicted to be resistant to two first-line drugs, and three (4.8%) with strains predicted to be resistant to one first-line drug. Predicted resistance was linked to mutations in *rpoB, inhA, rrs, and pncA*, and occurred across multiple Mtb lineages and in *M. bovis* isolates (Supplementary Table S1).

Treatment outcome data were available for all cases. Thirty-eight (58.5%) patients were cured, seven (10.8%) died during treatment, 20 (30.8%) were lost to follow-up (LTFU; Table 1). Of the seven patients who died during treatment, all were newly registered cases and received the standard first-line regimen (2HRZE/4HR). All seven corresponding isolates were predicted to be fully susceptible (Supplementary Table S1). Of note, all three patients with *M. bovis* infection were among those LTFU.

## 4 Discussion

The study provides the first comprehensive WGS analysis of MTBC isolates in Fiji, integrating genomic, epidemiological, and clinical data. Among 65 sequenced isolates collected between 2019 and 2022, most were Mtb, with a small proportion identified as *M. bovis*. The population structure was dominated by L4, followed by L3, L2, and L1. Although L4 was the predominant lineage, L3 formed the largest genomic cluster, with evidence of household-, community-, church-, and school-associated transmission, suggesting ongoing, recent localised transmission. Most isolates were drug susceptible, although a small proportion showed mono or multidrug resistance. While most cases received standard first-line treatment and were cured, deaths and substantial LTFU remained major challenges for TB control in Fiji. These findings indicate a genetically diverse MTBC population with multiple localized transmission networks rather than a single dominant outbreak strain, highlighting the importance of integrating genomic surveillance into TB control strategies in Fiji and the wider Pacific region.

The genomic analysis identified distinct transmission patterns across the major Mtb lineages circulating in Fiji. L4 remained the predominant lineage, consistent with its widespread distribution across Oceania (Mulholland et al., 2019; Yang et al., 2017; Stimson et al., 2019), whereas L3 demonstrated the largest and most epidemiologically interconnected transmission network. Unlike the smaller L1 and most L4 clusters, which were largely confined to household or neighbour-associated exposure, L3 clusters extended beyond household settings and involved schools, non-household close contacts, neighbouring communities, and interdivision travel. One L4 cluster also demonstrated possible church-associated transmission, further highlighting the role of communal social environments in TB spread. Although L2 is globally associated with increased transmissibility and drug resistance, only limited household-linked clustering was observed in this study. To our knowledge, this is the first report of Mtb L3 in a Pacific Island country, highlighting the emergence of a previously unrecognized lineage in the region. These findings are particularly important in the Fijian context, where strong communal connectivity, frequent social interaction, and population mobility may facilitate transmission beyond household settings alone. The identification of transmission networks spanning schools, churches, households, and neighbouring communities suggests that routine household contact tracing alone may be insufficient to interrupt ongoing transmission. Similar findings have been reported in other high-burden settings, where community and congregate environments contribute substantially to sustained TB transmission (Stucki et al., 2016; Brynildsrud et al., 2018). Together, these findings support expanding public health interventions beyond household-based investigations to include community screening, targeted active case finding, school and church-associated investigations, and integration of WGS into routine TB surveillance to enable earlier identification and interruption of transmission networks.

Treatment interruption remains a major challenge for TB control because it contributes to ongoing transmission, relapse, poor clinical outcomes, and the emergence of drug-resistant TB (Yanagawa et al., 2023). Although Fiji’s TB program utilizes both directly observed therapy (DOTS) and self-administered therapy (SAT; Yanagawa et al., 2024), 30.8% of cases in this study were LTFU after initiating treatment, indicating persistent gaps in treatment retention. Fiji’s 30.8% LTFU rate is higher than rates reported in some Pacific settings but comparable to others facing similar challenges (Yanagawa et al., 2023; Yanagawa et al., 2024; Charles et al., 2024). Notably, almost half of the cases with predicted drug-resistant isolates were either LTFU or died during treatment, raising concern that interrupted treatment may contribute to future amplification and spread of drug-resistant TB within the community. Moreover, almost 50% of LTFU cases originated from rural or outer island settings, suggesting that geographic isolation and healthcare accessibility may substantially influence treatment continuity. The high LTFU rate likely reflects multiple interacting challenges, including geographic isolation, inter-island travel, population mobility, socioeconomic hardship, stigma, treatment fatigue, limited long-term treatment support, and catastrophic TB-related costs, which affect approximately 45% of TB-affected households in Fiji (World Health Organization, 2025). Similar associations between financial hardship and poor treatment adherence have been reported in other high-burden settings (Aung et al., 2021). Across the Pacific, treatment outcomes vary considerably. Papua New Guinea reported a treatment success rate of 64%, with 19% of patients LTFU, reflecting challenges associated with difficult terrain, limited healthcare infrastructure, workforce shortages and barriers to accessing treatment services (Aia et al., 2018). In contrast, Solomon Islands achieved treatment success exceeding 90%, with LTFU declining to 0.3% following strengthening of its National TB Programme through decentralised service delivery, enhanced treatment monitoring and community-based patient support (Yanagawa et al., 2024). Similar high treatment success has also been reported in Tonga, Vanuatu, Palau and the Cook Islands, demonstrating the benefits of well-supported TB programmes across the Pacific (Yanagawa et al., 2023; Yanagawa et al., 2024; Charles et al., 2024). These findings, together with evidence from other resource-limited settings, suggest that strengthening decentralised TB services through patient-centred interventions, including enhanced case management, community health worker programmes, home-based treatment supervision, mobile health reminders, video-observed therapy, and targeted social and financial support, could improve treatment adherence and retention in Fiji (Mulaku et al., 2024; Heuvelings et al., 2017).

Drug resistance was uncommon, with most isolates predicted to be susceptible to first-line anti-TB drugs, consistent with previous reports from Fiji (Yanagawa et al., 2023; World Health Organization, 2022). A small number of isolates demonstrated resistance to rifampicin, streptomycin, and pyrazinamide, with resistance-associated mutations. Resistance was identified across multiple lineages, suggesting independent emergence rather than dissemination of a single dominant resistant clone. However, the low percentage of drug-resistant Mtb limited our ability to understand the resistance transmission dynamics. Although most genomic clusters involved drug-susceptible isolates, the presence of resistance-associated mutations across genetically diverse strains highlights the potential for resistant isolates to emerge and spread through existing household and community transmission networks if not detected early. Earlier detection of drug resistance could facilitate timely optimisation of treatment regimens, potentially improving patient outcomes and reducing onward transmission of resistant strains. These findings are particularly important given Fiji’s recent transition from an upper moderate TB incidence category to an endemic TB setting, alongside rising HIV cases and increasing evidence of ongoing community transmission (World Health Organization, 2025).

An important finding of this study was the identification of *M. bovis* among human TB cases. *M. bovis* is primarily associated with zoonotic transmission through infected cattle or consumption of contaminated animal products, although person-to-person airborne transmission has also been reported (Evans et al., 2007; Buss et al., 2014). Current routine diagnostic methods in Fiji do not distinguish *M. bovis* from Mtb, resulting in all three *M. bovis* cases initially receiving the standard first-line treatment regimen including pyrazinamide, which is not recommended for confirmed *M. bovis* infection because of its intrinsic resistance (del Valle et al., 2015). This may contribute to suboptimal treatment and potentially contribute to poor treatment outcomes. Of note, all three *M. bovis* cases were LTFU. The finding is particularly concerning given increasing reports of bovine TB in cattle in Fiji, which may increase opportunities for zoonotic transmission (Garcia et al., 2022; Toribio et al., 2023). These observations highlight the importance of species-level diagnostics and support the need for a One Health approach integrating human, veterinary, and agricultural surveillance systems. Despite WGS provides the highest resolution for species identification and genomic characterization (Nyunt et al., 2016; Mulholland et al., 2022; Mulholland et al., 2019), more affordable molecular approaches such as loop-mediated isothermal amplification (LAMP)-based assays may offer practical alternatives for differentiating *M. bovis* from Mtb in resource-limited settings (Jagielski et al., 2014; Akapelwa et al., 2025). While these methods do not provide the transmission and drug resistance information generated by WGS, they could improve species-level diagnosis and strengthen TB surveillance in countries where routine WGS capacity is not yet widely available.

Several limitations should be considered when interpreting these findings. First, only 10.6% of total laboratory confirmed cases were included in this study, which may limit the representativeness of the genomic dataset. As isolates were selected opportunistically rather than through systematic sampling, some transmission networks, lineage diversity, or resistance profiles may have been underrepresented, and the findings may not fully reflect the molecular epidemiology of TB across Fiji. Moreover, because information on the total number of archived isolates available during the study period was unavailable, the extent of potential selection bias could not be formally quantified. Future prospective genomic surveillance with systematic sampling and higher sequencing coverage would provide a more comprehensive understanding of national transmission dynamics and population structure. Second, although epidemiological data were obtained from patient records, the PATIS, and contact tracing reports, the completeness of epidemiological information varied between cases, limiting confirmation of transmission links for some genomic clusters despite close genomic relatedness. Consequently, the absence of documented epidemiological links may reflect incomplete investigations or previously undetected community transmission. Improved integration of epidemiological investigations with routine genomic surveillance would strengthen outbreak detection, transmission mapping, and interpretation of transmission dynamics. Third, phenotypic drug susceptibility testing was not performed, and resistance profiles were inferred solely from genomic mutations. Although WGS provides high-resolution prediction of resistance, the absence of phenotypic confirmation may reduce confidence for certain drugs or mutations. Integrating genomic and phenotypic drug susceptibility testing would improve the interpretation of resistance profiles and strengthen laboratory capacity for routine TB surveillance in Fiji. Fourth, no isolates were collected in 2020 because of disruptions associated with the COVID-19 pandemic, including diversion of laboratory resources and reduced diagnostic capacity. This temporal gap limits our ability to determine whether the genomic transmission clusters identified were maintained throughout the pandemic or were established primarily before it. Moreover, the study could not evaluate the potential influence of pandemic-related disruptions to healthcare access and TB case detection on transmission dynamics.

Finally, isolates were collected from selected years and may not fully capture longer-term temporal trends in lineage distribution and drug resistance across Fiji. Moreover, the small sample limited a robust evaluation of factors associated with poor treatment outcomes, including loss to follow-up. Information on the reasons for LTFU, such as transfer of care or treatment interruption, was also not available. Information distinguishing LTFU from undocumented transfer of care, treatment interruption, or other reasons was also not consistently available. Future prospective studies with comprehensive clinical follow-up are needed to better understand the determinants of treatment outcomes and their impact on TB transmission in Fiji.

In conclusion, this study provides the first genomic epidemiological characterization of MTBC in Fiji. It demonstrates a genetically diverse MTBC population with multiple localized transmission networks involving households, schools, churches, neighbouring communities, and interdivision movement. Although most isolates were drug susceptible, the detection of resistance-associated mutations across multiple lineages highlights the ongoing risk of emerging drug-resistant TB in Fiji. The identification of *M. bovis* among human TB cases further emphasizes the importance of zoonotic transmission and the need for integrated One Health surveillance approaches. High rates of LTFU, particularly among patients from rural and outer island settings, indicate persistent healthcare access and treatment retention challenges that may contribute to continued transmission and poor clinical outcomes. Collectively, these findings demonstrate the value of integrating genomic, epidemiological, and clinical investigations to strengthen TB surveillance, improve understanding of transmission dynamics, and guide targeted public health interventions. Establishing routine genomic surveillance capacity in Fiji may support earlier outbreak detection, improved monitoring of transmission and drug resistance, and strengthened TB control efforts across Fiji and the wider Pacific region.

## Data Availability

The datasets presented in this study can be found in online repositories. The names of the repository/repositories and accession number(s) can be found in the article/Supplementary material.

## Supplementary material

SUPPLEMENTARY FIGURE S1 Health administrative divisions of Fiji Map. Health services in Fiji are delivered under the Ministry of Health and Medical Services (MoHMS), which is organized into four administrative divisions: Central, Western, Northern, and Eastern. These divisions are further subdivided into smaller administrative units, including subdivisions, medical areas, and zones. Microbiological culture services are available at three divisional hospitals: Colonial War Memorial Hospital, Lautoka Hospital, and Labasa Hospital. Primary healthcare is provided through a network of 19 subdivisional hospitals and 189 peripheral health facilities. Private healthcare services are limited and predominantly located in urban centres. The MoHMS also includes Tamavua Twomey Hospital in Suva, which houses the Daulako Mycobacterium Reference Laboratory, the national reference laboratory for tuberculosis in Fiji, and plays a central role in laboratory-based TB surveillance. Map of Fiji showing the four administrative divisions: Central, Western, Northern, and Eastern. Source: Netmaps, Fiji Political Map.

SUPPLEMENTARY TABLE S1 Demographic, genomic, drug resistance, treatment, and clinical outcome characteristics of Mycobacterium tuberculosis complex isolates from Fiji, 2019, 2021-2022.
