# Supplementary material for: Genomic epidemiology of the Mycobacterium tuberculosis complex in Fiji reveals community transmission and human Mycobacterium bovis infection
Source: Front Microbiol. 2026 Aug 11;17:1933597. doi: 10.3389/fmicb.2026.1933597 (PMC13503548; doi:10.3389/fmicb.2026.1933597)
Supplement: Supplementary file 1 [file Data_Sheet_1.PDF]

Figure S1

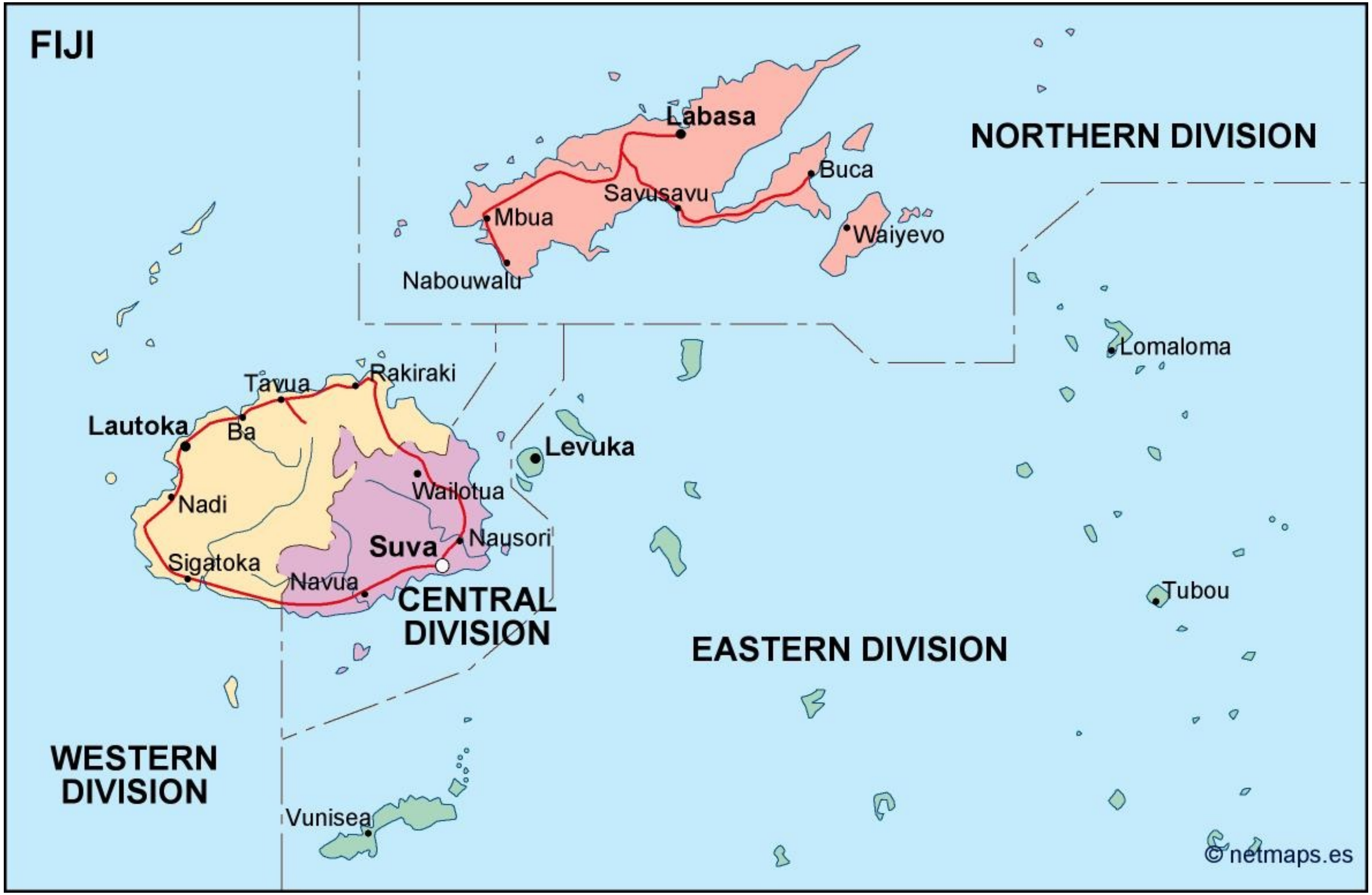
FIJI
NORTHERN DIVISION
Labasa
Buca
Savusavu
Mbua
Waiyevo
Nabouwalu
Lomaloma
Rakiraki
Tavua
Lautoka
Ba
Levuka
Wailotua
Nadi
Nausori
Suva
Sigatoka
Navua
CENTRAL DIVISION
EASTERN DIVISION
Tubou
WESTERN DIVISION
Vunisea
© netmaps.es
