# Supplementary material for: Genomic epidemiology of the Mycobacterium tuberculosis complex in Fiji reveals community transmission and human Mycobacterium bovis infection
Source: Front Microbiol. 2026 Aug 11;17:1933597. doi: 10.3389/fmicb.2026.1933597 (PMC13503548; doi:10.3389/fmicb.2026.1933597)
Supplement: Supplementary file 2 [file Data_Sheet_2.PDF]

	FJ-TB	COLLECTION YEAR	DIVISION	Age	GENDER	ETHNICITY	LINEAGE	SUB-LINEAGE
1	1	2021	Central	71	Male	iTaukei	Lineage 4	L4.1.1.2
2	2	2019	Eastern	64	Male	iTaukei	Lineage 1	L1.1.2
3	3	2019	Western	55	Male	Others	Lineage 2	L2.2.1
4	4	2022	Central	35	Female	Indo-Fijian	Lineage 1	L1.1.2
5	5	2019	Central	2	Male	iTaukei	Lineage 4	L4.3.3
6	6	2019	Central	29	Female	iTaukei	Lineage 3	L3
7	7	2019	Central	62	Female	iTaukei	Lineage 4	L4.4.1.1
8	8	2022	Western	64	Male	iTaukei	Lineage 4	L4.3.3
9	9	2022	Central	61	Male	Others	Lineage 2	L2.2.1
10	10	2019	Central	29	Male	iTaukei	Lineage 4	L4.3.3
11	11	2021	Central	25	Male	iTaukei	M.bovis	M.bovis
12	13	2021	Central	19	Female	iTaukei	Lineage 2	L2.2.1.1
13	14	2019	Central	21	Female	iTaukei	Lineage 4	L4.3.3
14	15	2021	Central	24	Female	iTaukei	Lineage 3	L3
15	18	2019	Central	23	Male	iTaukei	Lineage 4	L4.3.3
16	19	2022	Central	35	Male	iTaukei	Lineage 4	L4.1.1.2
17	20	2021	Central	19	Female	iTaukei	Lineage 2	L2.2.1
18	21	2022	Central	35	Female	iTaukei	Lineage 4	L4.3.3
19	22	2021	Western	20	Male	Others	Lineage 4	L4.1.1.3
20	23	2022	Central	36	Male	iTaukei	Lineage 2	L2.2.1
21	24	2021	Western	21	Male	iTaukei	Lineage 2	L2.2.1.1
22	25	2022	Central	54	Female	iTaukei	Lineage 4	L4.3.3
23	26	2019	Northern	59	Female	Indo-Fijian	Lineage 1	L1.1.2
24	27	2019	Central	41	Female	iTaukei	Lineage 4	L4.3.3
25	28	2022	Western	58	Female	Indo-Fijian	Lineage 1	L1.2.2.2
26	29	2022	Eastern	73	Female	iTaukei	Lineage 4	L4.3.3
27	30	2019	Central	27	Female	iTaukei	Lineage 4	L4.4.1.1
28	31	2019	Western	32	Female	iTaukei	Lineage 4	L4.7
29	32	2019	Central	79	Male	Others	Lineage 4	L4.3.3
30	33	2021	Western	25	Male	iTaukei	Lineage 3	L3
31	34	2021	Western	26	Male	iTaukei	Lineage 2	L2.2.1
32	35	2022	Central	22	Female	iTaukei	Lineage 4	L4.3.3
33	36	2022	Eastern	73	Female	iTaukei	Lineage 4	L4.3.3

34	37	2019	Central	26	Female	iTaukei	Lineage 3	L3
35	38	2022	Central	14	Female	iTaukei	Lineage 4	L4.3.3
36	39	2019	Central	44	Female	iTaukei	Lineage 3	L3
37	40	2021	Central	46	Male	Indo-Fijian	Lineage 4	L4.3.3
38	41	2021	Western	53	Male	Others	Lineage 1	L1.1.2
39	42	2022	Northern	62	Male	iTaukei	Lineage 3	L3
40	44	2019	Central	32	Female	iTaukei	Lineage 1	L1.1.2
41	45	2021	Central	29	Male	iTaukei	Lineage 2	L2.2.1.1
42	46	2019	Central	23	Female	iTaukei	Lineage 3	L3
43	47	2019	Northern	30	Female	iTaukei	Lineage 3	L3
44	48	2019	Western	58	Male	Indo-Fijian	Lineage 1	L1.1.2
45	49	2019	Western	25	Male	iTaukei	M.bovis	M.bovis
46	50	2022	Central	27	Male	iTaukei	Lineage 1	L1.1.2
47	51	2019	Northern	18	Female	iTaukei	Lineage 3	L3
48	53	2019	Central	9	Female	iTaukei	Lineage 3	L3
49	54	2022	Central	16	Female	Others	Lineage 2	L2.2.1.1
50	55	2019	Eastern	32	Male	iTaukei	Lineage 4	L4.3.3
51	56	2019	Central	67	Female	iTaukei	Lineage 4	L4.3.3
52	57	2022	Eastern	31	Female	iTaukei	Lineage 2	L2.2.1
53	58	2019	Central	52	Female	iTaukei	Lineage 4	L4.3.3
54	60	2019	Eastern	68	Male	iTaukei	Lineage 4	L4.3.3
55	61	2019	Central	45	Male	iTaukei	M.bovis	M.bovis
56	62	2019	Western	39	Male	iTaukei	Lineage 4	L4.3.3
57	63	2019	Western	66	Male	iTaukei	Lineage 4	L4.7
58	64	2022	Central	36	Male	iTaukei	Lineage 3	L3
59	65	2019	Northern	57	Female	Indo-Fijian	Lineage 1	L1.1.2
60	66	2019	Central	39	Female	iTaukei	Lineage 2	L2.2.2
61	67	2022	Western	23	Male	iTaukei	Lineage 4	L4.3.3
62	68	2019	Eastern	34	Male	iTaukei	Lineage 3	L3
63	79	2019	Central	31	Male	iTaukei	Lineage 4	L4.3.3
64	81	2022	Eastern	61	Female	iTaukei	Lineage 4	L4.8
65	82	2019	Western	63	Female	iTaukei	Lineage 3	L3

PREDICTED DRUG RESISTANCE	RESISTANCE	SUPPORTING MUTATIONS	Treatment hist	Treatment (Drugs) is	Tretment outcome
Sensitive			New	2 HRZE 4 HR	Cured
Sensitive			New	2 HRZE 4 HR	Cured
Sensitive			New	2 HRZE 4 HR	Lost to follow up
Sensitive			New	2 HRZE 4 HR	Lost to follow up
Sensitive			New	2 HRZE 4 HR	Cured
Sensitive			New	2 HRZE 4 HR	Cured
Sensitive			New	2 HRZE 4 HR	Cured
Sensitive			New	2 HRZE 4 HR	Cured
Sensitive			Relapse	2 HRZE 4 HR	Cured
Sensitive			New	2 HRZE 4 HR	Cured
Pyrazinamide	*pncA*	p.His57Asp	New	2 HRZE 4 HR	Lost to follow up
Sensitive			New	2 HRZE 4 HR	Cured
Sensitive			New	2 HRZE 4 HR	Lost to follow up
Sensitive			New	3 HRZE 5 HR	Lost to follow up
Sensitive			New	2 HRZE 4 HR	Cured
Sensitive			New	2 HRZE 4 HR	Cured
Isoniazid, Pyrazinamide, Streptomycin	*katG, pncA, rrs*	p.Ser315Thr ,p.Gln141Pro, n.514A>C	New	2 HRZE 4 HR	Lost to follow up
Isoniazid, Ethionamide	*inhA*	c.-777C>T	New	2 HRZE 4 HR	Lost to follow up
Sensitive			New	2 HRZE 4 HR	Lost to follow up
Isoniazid, Pyrazinamide, Streptomycin	*katG, pncA, rrs*	p.Ser315Thr ,p.Gln141Pro, n.514A>C	New	2 HRZE 4 HR	Lost to follow up
Sensitive			New	2 HRZE 4 HR	Cured
Sensitive			New	2 HRZE 4 HR	Cured
Sensitive			New	2 HRZE 4 HR	Deceased
Sensitive			New	2 HRZE 4 HR	Cured
Sensitive			New	2 HRZE 4 HR	Cured
Sensitive			New	2 HRZE 4 HR	Lost to follow up
Sensitive			New	2 HRZE 4 HR	Lost to follow up
Sensitive			New	2 HRZE 4 HR	Cured
Sensitive			New	2 HRZE 4 HR	Deceased
Sensitive			New	2 HRZE 4 HR	Cured
Sensitive			New	2 HRZE 4 HR	Cured
Sensitive			New	2 HRZE 4 HR	Lost to follow up
Sensitive			New	2 HRZE 4 HR	Lost to follow up

Sensitive			New	2 HRZE 4 HR	Cured
Sensitive			New	2 HRZE 4 HR	Deceased
Sensitive			New	2 HRZE 4 HR	Cured
Sensitive			New	2 HRZE 4 HR	Cured
Sensitive			New	2 HRZE 4 HR	Deceased
Sensitive			New	2 HRZE 4 HR	Cured
Sensitive			New	2 HRZE 4 HR	Cured
Sensitive			New	2 HRZE 4 HR	Cured
Sensitive			New	2 HRZE 4 HR	Cured
Sensitive			New	2 HRZE 4 HR	Deceased
Sensitive			New	2 HRZE 4 HR	Cured
Pyrazinamide	*pncA*	p.His57Asp	New	2 HRZE 4 HR	Lost to follow up
Rifampicin	*rpoB*	p.His445Leu	New	2 HRZE 4 HR	Lost to follow up
Sensitive			New	2 HRZE 4 HR	Cured
Sensitive			New	2 HRZE 4 HR	Cured
Rifampicin	*rpoB*	p.His445Leu	New	SLD	Cured
Sensitive			New	2 HRZE 4 HR	Lost to follow up
Sensitive			New	2 HRZE 4 HR	Deceased
Sensitive			New	2 HRZE 4 HR	Cured
Sensitive			New	2 HRZE 4 HR	Cured
Sensitive			New	2 HRZE 4 HR	Cured
Pyrazinamide	*pncA*	p.His57Asp	New	2 HRZE 4 HR	Lost to follow up
Sensitive			New	2 HRZE 4 HR	Cured
Sensitive			New	2 HRZE 4 HR	Lost to follow up
Sensitive			New	2 HRZE 4 HR	Lost to follow up
Sensitive			New	2 HRZE 4 HR	Deceased
Sensitive			New	2 HRZE 4 HR	Lost to follow up
Sensitive			New	2 HRZE 4 HR	Lost to follow up
Sensitive			New	2 HRZE 4 HR	Cured
Sensitive			New	2 HRZE 4 HR	Lost to follow up
Sensitive			New	2 HRZE 4 HR	Cured
Sensitive			New	2 HRZE 4 HR	Cured
